# Overview of Five-Years of Experience Performing Non-Invasive Fetal Sex Assessment in Maternal Blood

**DOI:** 10.3390/diagnostics3020283

**Published:** 2013-05-15

**Authors:** Sara Perlado-Marina, Ana Bustamante-Aragones, Laura Horcajada, Maria Jose Trujillo-Tiebas, Isabel Lorda-Sanchez, Marta Ruiz Ramos, Javier Plaza, Marta Rodriguez de Alba

**Affiliations:** 1 Genetics Department, Fundacion Jimenez Diaz, Avda, Reyes Catolicos 2, Madrid 28040, Spain; E-Mails: sara.perlado@fjd.es (S.P.-M.); abustamante@fjd.es (A.B.-A.); laura.horcajada@fjd.es (L.H.); mjtrujillo@fjd.es (M.J.T.-T.); ilorda@fjd.es (I.L.-S.); martaruizramos@hotmail.com (M.R.R.); 2 Centre for Biomedical Network Research on Rare Diseases (CIBERER), ISCIII, Valencia 46010, Spain; 3 Gynecology and Obstetric Department, Fundacion Jimenez Diaz, Avda, Reyes Catolicos 2, Madrid 28040, Spain; E-Mail: jplaza@fjd.es

**Keywords:** non-invasive prenatal diagnosis, fetal DNA, maternal plasma, fetal sex determination

## Abstract

Since the discovery of the presence of fetal DNA in maternal blood, non-invasive fetal sex determination has been the test most widely translated into clinical practice. To date there is no agreement between the different laboratories performing such tests in relation to which is the best protocol. As a consequence there are almost as many protocols as laboratories offering the service, using different methodologies and thus obtaining different diagnostic accuracies. By the end of 2007, after a validation study performed in 316 maternal samples collected between the 5th and 12th week of gestation, the fetal sex determination was incorporated into clinical practice in our Service. The test is performed in the first trimester of pregnancy, and it is offered as part of the genetic counseling process for couples at risk of X-linked disorders. As a general rule and in order to avoid misdiagnosis, two samples at different gestational ages are tested per patient. The analysis is performed by the study of the *SRY* gene by RT-PCR. Two hundred and twenty six pregnancies have been tested so far in these 5 years. Neither false positives nor false negatives diagnoses have been registered, thus giving a diagnostic accuracy of 100%.

## 1. Introduction

Lo’s discovery in 1997 of the presence of cell-free fetal DNA circulating (ccffDNA) in maternal blood by means of the detection of Y chromosome sequences in male bearing pregnancies was a milestone not only for the discovery itself, but also because it opened the door to the first non-invasive diagnosis that has been incorporated into clinical practice [1,2,3,4]. 

Fetal sexing is nowadays performed by many laboratories worldwide and it is one of the crucial steps in the management of pregnancies at risk of an X-linked disorder, since it may mean avoiding invasive prenatal diagnosis (PD) [5]. In fact this test has been introduced as an essential step in the management of pregnancies at risk of an X-linked disorder since fetal sex determination reduces the need for subsequent invasive testing only to those male-bearing pregnancies [6,7,8]. Because of prenatal DNA, studies are mainly carried out between the 10th and 12th weeks of gestation by chorion villus biopsy (CVS); non invasive sex determination ideally should be carried out in the first trimester of pregnancy. Generally, the main concerns regarding this test are the false positive and false negative results. False positives usually result from contamination, although, theoretically, they could also result from a vanishing twin or confined placental mosaicism. Nevertheless, they do not represent a risk for the fetus to be born with the disease since it would be tested by conventional PD. On the other hand, a false negative result may have important clinical consequences since the need for obstetric invasive procedures would then be discarded. Usually, laboratories performing such tests ask for ultrasound scan confirmation, which is more accurate in the 2nd than in the 1st trimester of gestation, thus meaning that, in case of a male fetus, an amniocentesis would be needed to figure out the affected/non-affected condition of the fetus. In spite of this critical aspect of the test, to date there is no agreement between the different laboratories performing such tests in relation to which is the best protocol. As a consequence there are almost as many protocols as laboratories offering the service, using different methodologies, diagnostic algorithms, scoring criteria and thus obtaining different diagnostic accuracies [9,10,11,12,13,14,15,16,17,18]. A recent systematic review and meta-analysis concluded that this test is highly accurate—it has been reported to be higher than 95% by several groups—and useful in clinical practice [19].

By the end of 2007, after a validation study performed in 316 maternal samples collected between the 5th and 12th weeks of gestation, the non-invasive fetal sex determination was incorporated into clinical practice in our Service [5]. The test is performed in the first trimester of pregnancy, and it is offered as part of the genetic counseling process for couples at risk of X-linked disorders. As a general rule and in order to avoid misdiagnosis, two samples at different gestational ages are tested per patient. The analysis is performed by the study of the *SRY* gene by RT-PCR. Two hundred and twenty six pregnancies have been tested so far in these 5 years. Neither false positives nor false negatives diagnoses have been registered, thus giving a diagnostic accuracy of 100%.

## 2. Experimental Section

### 2.1. Sample Collection

**Figure 1:**
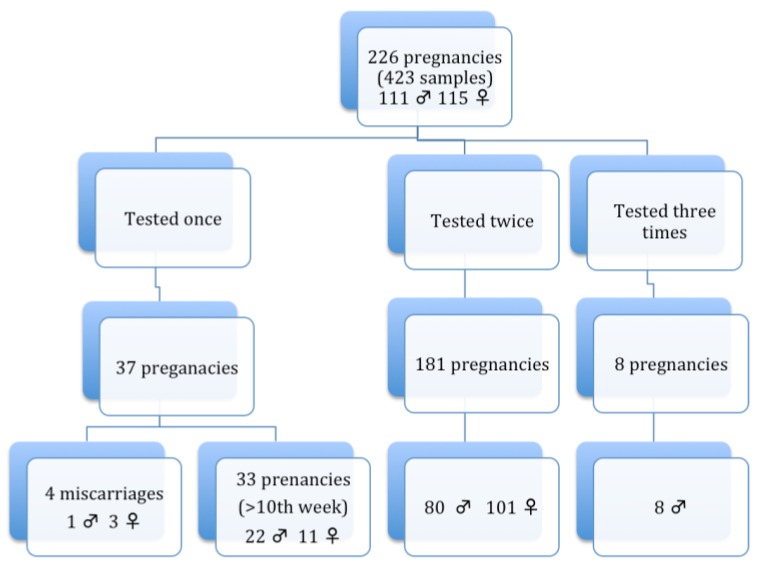
Schematic representation of the 226 pregnancies studied over 5 years.

From September 2007 to December 2012, 226 pregnant women have been tested, comprising a total of 30 different X-linked disorders (Figure 1; Table 1). All pregnant women are requested to have and ultrasound scan prior to blood sampling in order to date pregnancy. The minimum gestational age should be 7 weeks [5]. Ideally, in each pregnancy two samples per patient are studied, the first one in the 7th–8th weeks and the second 2 weeks later. Exceptionally, some cases have been studied at advanced gestational ages (10th–29th) mainly because of a delayed pregnancy control or ambiguous genitalia by sonographic scan. In these cases, only one sample was tested due to the presence of larger amounts of fetal DNA at these gestational ages and the need for a quick result. Pregnancies in which a discrepancy between both samples is detected are asked for a third one. All samples are collected after proper Genetic Counseling.

**Table 1:** Referral reason for sex determination in the 226 pregnancies studied (number of cases).

Haemophilia (93)	Incontinentia Pigmenti (2)
Duchenne/Becker Muscular Distrophy (42)	X-linked Cardiomyopathy (1)
Congenital Adrenal Hyperplasia (9)	X-linked Mental Retardation (1)
Hypophosphatemic dysplasia (6)	Morris Syndrome (1)
Norrie Syndrome (6)	Ambiguous Genitalia (3)
Agammaglobulinemia (6)	Menkes Disease (2)
X-linked Retinitis Pigmentosa (6)	X-linked Deafness (1)
X-linked inmunodeficiency (5)	Alport Syndrome (1)
Fragile X (6)	X-linked Tubular Myopathy (1)
PGD Confirmation (5)	Lowe Syndrome (1)
Retinoschisis (6)	Bruton Syndrome (1)
Kennedy Disease (4)	Androgen insensitivity syndrom (1)
X-linked Ocular Albinism (4)	Barth Syndrome (1)
Adenoleucodistrophy (4)	X-linked Fabry Disease (1)
Hunter Syndrome (5)	Ornitiltranscarbamilasa Deficiency (1)

### 2.2. Sample Processing and DNA Extraction

Twenty milliliters of maternal peripheral blood are collected in tubes with ethylene diamine tetra-acetic acid (EDTA) anticoagulant. Processing is preferably carried out by female personnel to avoid contamination with male genetic material. Specimens are centrifuged at 1,600 ×g for 10 min at room temperature in the original collection tube. Plasma is collected, divided in 1 mL aliquots and centrifuged at 13,000 ×g for 10 min. The supernatant is removed from each aliquot and placed in new collection tubes and stored at −20 °C. An aliquot of 1 mL of plasma sample is used for DNA extraction.

Samples are thawed at room temperature and DNA is automatically extracted using the QIACube with QIAamp DNA blood Mini Kit (Qiagen Inc., Hilden, Germany) according to the protocol previously described [20]. DNA is eluted in 50 μL of elution buffer (provided in the kit). Recently we have incorporated an additional extraction method: the MagNA Pure Compact Nucleic Acid Isolation Kit I-Large Volume (Roche, Mannheim, Germany) using the MagNA Pure equipment (Roche). Results are similar with both extraction DNA methods.

### 2.3. Real-Time PCR

Fetal sex assessment is based on detecting the presence of the sex-determining region of the Y chromosome (*SRY* gene). In addition, the glyceraldehyde phosphate dehydrogenase gene (*GAPDH *gene) is used as internal control for the DNA extraction process and polymerase chain reactions (PCR). Both targets are analysed independently in singleplex reactions to prevent outcompetition of the *SRY* amplification. Each sample is tested three times for the *SRY* gene and twice for the *GAPDH* gene. In each replicate, 8 μL of DNA extracted from the plasma sample is used. A sample of male genomic DNA and a female genomic DNA is included in the PCR as controls. For these samples, only 1 μL of genomic DNA is 115 used for the PCR amplification. All reactions are carried out in a final volume of 25 μL. Reactions for the *SRY* amplification contain 300 nM of the forward primer (SRY-F: 5'-GCGACCCATGAACGCATT-3'), 900 nM of the reverse primer (SRY-R: 5'-GCCATCTTGCGCCTCTGA-3'), 200 nM of the Taqman probe (SRY-P: 6-FAM CGTGTGGTCTCGCG-MGB) and 12.5 μL of Taqman Universal PCR Master Mix (Applied Biosystems, Foster City, CA, USA). Reactions for the GAPDH amplification contain 300 nM of each primer (forward primer GAPDH-F: 5'-CCCCACACACATGCACTTACC-3' and reverse primer GAPDH-R: 5'-CCTAGTCCCAGGGCTTTGATT-3') and 200 nM of the Taqman probe (GAPDH-R: VIC-AAAGAGCTAGGAAGGACAGGCAACTTGGC- TAMRA) and 12.5 μL of Taqman Universal PCR Master Mix (Applied Biosystems). Thermal cycling was initiated with 2 min incubation at 50 °C, followed by a first denaturation phase at 95 °C for 10 min and then 40 cycles of 95 °C for 15 s and 60 °C for 1 min. All probes and primers are supplied by Applied Biosystems. Amplification data are collected and analysed with an ABI Prism 7000 Sequence Detection System (Applied Biosystems).

### 2.4. Scoring Model

The two collected samples are tested on different days, results registered and compared once the 2nd sample is analyzed. In pregnancies tested twice:

- for positive results (male fetus), only one replicate of the *SRY* gene is allowed to fail among replicates of both samples. Ct < 38 is considered as positive.
- for negative results (female fetus), only one positive result for 139 the *SRY* gene is allowed among replicates of both samples.

In pregnancies tested once no discrepancies between the three *SRY* replicated are allowed.

Because some of the centers that were sending samples for the first time were not aware of the timing for sampling, in eight pregnancies the first sample was collected before the 7th week of gestation. In these cases a third sample was required to confirm the diagnosis. 

## 3. Results and Discussion

### 3.1. Results

The median gestational age for the first sample was 8 weeks (ranging from 7–9) and for the second sample was 10 weeks (ranging from 9–11). As described above, some samples were collected under exceptional circumstances (before the 7th week and after the 10th) and so do not fulfill these criteria. Only 4 out of the 226 pregnancies tested no confirmation after the test could be done due to a spontaneous miscarriage. All 4 were only tested once, resulting in 3 females and 1 male. The remaining 222 pregnancies were correctly diagnosed. One hundred and eighty one pregnancies were tested twice, 33 only tested once and 8 three times. The mean Ct for the *SRY* and *GAPD*H genes were 35.91 (SD 1.20) and 30.31 (SD 1.81) respectively. A total of 111 males and 115 females were diagnosed. All female pregnancies (50%) avoided conventional prenatal diagnosis.

Confirmation methods: Two different confirmation methods were used, conventional prenatal diagnosis and ultrasound scan.

### 3.2. Discussion

It is widely accepted that non-invasive fetal sex determination based on the identification of ccffDNA in maternal blood provides a safe alternative to conventional invasive genetic studies of the fetus. Here we show how a simple protocol that implies the study of the *SRY* gene in two samples per pregnant women, with a time lapse of 2 weeks between them, allows us to have a 100% accuracy in the diagnosis. This level of certainty has been pursued by many groups using different methodologies, diagnostic algorithms, scoring criteria [9,10,11,12,13,14,15,16,17,18]. A recent systematic review and meta-analysis, on 57 selected studies, concluded that the overall sensitivity was 95.4% (95% confidence interval [CI], 94.7%–96.1%) and specificity 98.6% (95% CI, 0.989–0.995); meaning that this test is highly accurate useful in clinical practice [19]. However, those performing this test have major concerns regarding false positive and false negative results. Moreover, as previously mentioned, a false negative result may have important clinical consequences since the need for obstetric invasive procedures would then be discarded. In the case of false positives, the associated risk regards the invasive obstetric procedure. For this reason, new ways of performing the non-invasive sex assessment, looking for the highest accuracy are being described.

A diagnostic algorithm combining the results of *DYS14* and *SRY* polymerase chain reaction is the analysis most commonly used [8,11,18]. Generally, *DYS14* is firstly tested due to its highest sensitivity and those cases that tested positive are afterwards tested for *SRY* for confirmation to avoid false positives. However, evidence of positive results in female bearing pregnancies are reported and for this reason some groups have introduced another marker to the algorithm [13]. However, the addition of new markers together with the very stringent criteria that are commonly used, give result to a percentage of inconclusive results [11,18]. Rong *et al*. [15] have recently reported another strategy that uses a multiplex PCR of 17 different STR with which they claim 100% diagnostic accuracy, despite all STR not being amplified in any of the samples. Although their results were promising, the assay was carried out with samples from the 2nd and 3rd trimester of gestation, while this test should be done in the 1st trimester in order to avoid conventional prenatal diagnosis. In addition, multiplex PCR is more critical in the first trimester because of the low concentration of fetal DNA and the competition between primers. Kim *et al*. [14] have also reported their results using multiplex PCR for the *AMELX/Y* and *SRY* genes. The study compares the sensitivity and specificity obtained testing only for *SRY*, *AMELX/Y* and the combination of both. By combining the analysis of both genes they get the highest sensitivity and specificity but a false negative rate of 1.2%. Multiplex QF-PCR not only has the problem of competition between primers but also that it is less sensitive than RT-PCR. A small study performed in our laboratory in the plasma samples from the 1st trimester of pregnancy (data not shown) has demonstrated that the detection of the *SRY* gene can be done earlier in gestation by RT-PCR than with QF-PCR. In the absence of replicates for the *SRY* gene, and to make sure that the absence of Y chromosome is because the fetus is a female, other groups have designed new tests to prove the presence of fetal DNA in the maternal blood. The majority of them focus on the differences in methylation between fetal and maternal sequences [12,16] but others look for different strategies like searching for paternal polymorphisms in maternal blood [8]. These strategies have the only disadvantage that generally these studies are carried out by conventional PCR which is, as mentioned before, less sensitive than RT-PCR. Therefore, in case the presence of fetal DNA is not confirmed and the amplification for the Y sequences is negative, the result is considered inconclusive. Anyhow, the result obtained for the fetal sex assessment could be right. Scheffer *et al*. [8] reported 10 samples in which there was neither detection of the Y-chromosome nor paternal polymorphisms; they reported the result as inconclusive. However, the outcome of those cases showed that all fetuses were females. White *et al*. [16] tried to overcome this problem by using RT-PCR for both studies, *SRY* gene and differences in methylation. They documented that an incomplete digestion of the *RASSF1*A sequences was observed in plasma samples and that the protocol has to be modified to test maternal plasma samples. Though both assays have identical reaction conditions and show good results, they still find unreportable levels of hypermethylated *RASSF1A* together with positive *SRY* replicates.

Here, we have reported a simple diagnostic flow for fetal sex assessment that uses an automated system for DNA extraction and only tests for the *SRY* gene (and the *GAPDH* as internal control) and that has shown to be 100% accurate. Recently, new reports looking for certainty in the confirmation of the presence of fetal DNA in the maternal blood are describing new diagnostic algorithms that imply many different tests. However, the test is getting less simple and more expensive. From a clinical laboratory perspective, incorporation of this study in the daily routine should be effortless. Besides, for this specific analysis, it is important to avoid too much handling to avoid the chance of contamination. Collection of maternal blood samples based on sonographic gestational age and study of two independent samples reduces the risk of misdiagnosis. This is especially important to consider for those samples collected at early gestational ages (before the 9th week). In fact, there were 8 pregnancies in which we had to ask for a third sample for an accurate result. These cases were coming from other centers and sonographic confirmation of gestational age could not be done beforehand.

## 4. Conclusion

In conclusion, fetal sex assessment in maternal blood is at present an essential tool in the management of pregnancies at risk of X-linked disorders. This test has widely shown to be accurate from the 7th week of gestation. Although there is not a general consensus about the best algorithm to be followed, time consuming and expensive diagnostic approaches should be avoided in a clinical setting.

## References

[1] Lo Y.M., Corbetta N., Chamberlain P.F., Rai V., Sargent I.L., Redman C.W., Wainscoat J.S. (1997). Presence of fetal DNA in maternal plasma and serum. Lancet.

[2] Johnson K.L., Dukes K.A., Vidaver J., LeShane E.S., Ramirez I., Weber W.D., Bischoff F.Z., Hahn S., Sharma A., Dang D.X. (2004). Interlaboratory comparison of fetal male DNA detection from common maternal plasma samples by real-time PCR. Clin. Chem..

[3] Ho S.S., Damayanti Z., Chua W.Y., Ng B.L., Peh C.M., Biswas A., Choolani M. (2004). Non-invasive prenatal diagnosis of fetal gender using real-time polymerase chain reaction amplification of SRY in maternal plasma. Ann. Acad. Med. Singap..

[4] Tachdjian G., Frydman N., Audibert F., Ray P., Kerbrat V., Ernault P., Frydman R., Costa J.M. (2002). Clinical applications of fetal sex determination in maternal blood in a preimplantation genetic diagnosis centre. Hum. Reprod..

[5] Bustamante-Aragones A., Rodriguez de Alba M., Gonzalez-Gonzalez C., Trujillo-Tiebas J., Diego-Alvarez D., Vallespin E., Plaza J., Ayuso C., Ramos C. (2008). Foetal sex determination in maternal blood from the seventh week of gestation and its role in diagnosing haemophilia in the foetuses of female carriers. Haemophilia.

[6] Avent N.D., Chitty L.S. (2006). Non-invasive diagnosis of fetal sex; utilisation of free fetal DNA in maternal plasma and ultrasound. Prenat. Diagn..

[7] Finning K.M., Chitty L.S. (2008). Non-invasive fetal sex determination: Impact on clinical practice. Semin. Fetal Neonatal Med..

[8] Scheffer P.G., van der Schoot C.E., Page-Christiaens G.C., Bossers B., van Erp F., de Haas M. (2010). Reliability of fetal sex determination using maternal plasma. Obstet. Gynecol..

[9] Akolekar R., Farkas D.H., van Agtmael A.L., Bombard A.T., Nicolaides K.H. (2010). Fetal sex determination using circulating cell-free fetal DNA (ccffDNA) at 11 to 13 weeks of gestation. Prenat. Diagn..

[10] Miura K., Higashijima A., Shimada T., Miura S., Yamasaki K., Abe S., Jo O., Kinoshita A., Yoshida A., Yoshimura S. (2011). Clinical application of fetal sex determination using cell-free fetal DNA in pregnant carriers of X-linked genetic disorders. J. Hum. Genet..

[11] Hill M., Finning K., Martin P., Hogg J., Meaney C., Norbury G., Daniels G., Chitty L.S. (2011). Non-invasive prenatal determination of fetal sex: Translating research into clinical practice. Clin. Genet..

[12] Lim J.H., Park S.Y., Kim S.Y., Kim D.J., Choi J.E., Kim M.H., Choi J.S., Kim M.Y., Yang J.H., Ryu H.M. (2012). Effective detection of fetal sex using circulating fetal DNA in first-trimester maternal plasma. FASEB J..

[13] Fernández-Martínez F.J., Galindo A., Garcia-Burguillo A., Vargas-Gallego C., Nogués N., Moreno-García M., Moreno-Izquierdo A. (2012). Non inavsive fetal sex determination in maternal plasma: A prospective feasibility study. Genet. Med..

[14] Kim S.Y., Lim J.H., Park S.Y., Kim M.Y., Choi J.S., Ryu H.M. (2012). Non-invasive prenatal determination of fetal gender using QF-PCR analysis of cell-free fetal DNA in maternal plasma. Clin. Chim. Acta.

[15] Rong Y., Gao J., Jiang X., Zheng F. (2012). Multiplex PCR for 17 Y-chromosome specific short tandem repeats (STR) to enhance the reliability of fetal sex determination in maternal plasma. Int. J. Mol. Sci..

[16] White H.E., Dent C.L., Hall V.J., Crolla J.A., Chitty L.S. (2012). Evaluation of a novel assay for detection of the fetal marker RASSF1A: Facilitating improved diagnostic reliability of non-invasive prenatal diagnosis. PLoS One.

[17] Aghanoori M.R., Vafaei H., Kavoshi H., Mohamadi S., Goodarzi H.R. (2012). Sex determination using 291 free fetal DNA at early gestational ages: A comparison between modified mini-STR genotyping method and real-time PCR. AJOG.

[18] Kolialexi A., Tounta G., Apostolou P., Vrettou C., Papantoniou N., Kanavakis E., Antsaklis A., Mavrou A. (2012). Early non-invasive detection of fetal Y chromosome sequences in maternal plasma using multiplex PCR. Eur. J. Obstet. Gynecol. Reprod. Biol..

[19] Devaney S.A., Palomaki G.E., Scott J.A., Bianchi D.W. (2011). Noninvasive fetal sex determination using cell-free fetal DNA: A systematic review and meta-analysis. JAMA.

[20] Galbiati S., Smid M., Gambini D., Ferrari A., Restagno G., Viora E., Campogrande M., Bastonero S., Pagliano M., Calza S. (2005). Fetal DNA detection in maternal plasma throughout gestation. Hum. Genet..

